# Supplementary material for: Multi‐dataset identification of innovative feature genes and molecular mechanisms in keratoconus
Source: J Cell Mol Med. 2024 Sep 19;28(18):e70079. doi: 10.1111/jcmm.70079 (PMC11412914; doi:10.1111/jcmm.70079)
Supplement: Supplementary file 2 — Material S2. [file JCMM-28-e70079-s002.zip › 7.GSEA analysis/6.SNORA74B/KEGG_CYTOKINE_CYTOKINE_RECEPTOR_INTERACTION.html]

Details for gene set KEGG\_CYTOKINE\_CYTOKINE\_RECEPTOR\_INTERACTION[GSEA]

|  || Dataset | SNORA74B.group.cls#h\_versus\_l |
| Phenotype | group.cls#h\_versus\_l |
| Upregulated in class | l |
| GeneSet | KEGG\_CYTOKINE\_CYTOKINE\_RECEPTOR\_INTERACTION |
| Enrichment Score (ES) | -0.46019578 |
| Normalized Enrichment Score (NES) | -1.3701968 |
| Nominal p-value | 0.0407767 |
| FDR q-value | 0.34602994 |
| FWER p-Value | 0.918 |
Table: GSEA Results Summary

  

Fig 1: Enrichment plot: KEGG\_CYTOKINE\_CYTOKINE\_RECEPTOR\_INTERACTION      
 Profile of the Running ES Score & Positions of GeneSet Members on the Rank Ordered List

  

| SYMBOL | TITLE | RANK IN GENE LIST | RANK METRIC SCORE | RUNNING ES | CORE ENRICHMENT || 1 | IL4R | na | 620 | 0.563 | -0.0073 | No |
| 2 | PF4 | na | 941 | 0.497 | -0.0071 | No |
| 3 | LEP | na | 1168 | 0.461 | -0.0049 | No |
| 4 | IFNA14 | na | 1314 | 0.437 | -0.0008 | No |
| 5 | IL21 | na | 1500 | 0.416 | 0.0018 | No |
| 6 | IFNA4 | na | 1727 | 0.395 | 0.0027 | No |
| 7 | IL12A | na | 2028 | 0.368 | 0.0010 | No |
| 8 | IFNL3 | na | 2063 | 0.366 | 0.0070 | No |
| 9 | BMPR1B | na | 2240 | 0.352 | 0.0086 | No |
| 10 | IFNA6 | na | 2354 | 0.343 | 0.0118 | No |
| 11 | IL3RA | na | 2432 | 0.337 | 0.0160 | No |
| 12 | IL17B | na | 2567 | 0.328 | 0.0184 | No |
| 13 | IL26 | na | 2581 | 0.327 | 0.0242 | No |
| 14 | NGFR | na | 2599 | 0.326 | 0.0299 | No |
| 15 | AMHR2 | na | 2619 | 0.325 | 0.0355 | No |
| 16 | IL9 | na | 2712 | 0.319 | 0.0389 | No |
| 17 | TNFRSF25 | na | 3513 | 0.277 | 0.0210 | No |
| 18 | IFNA16 | na | 3517 | 0.277 | 0.0262 | No |
| 19 | TNFRSF13C | na | 3685 | 0.271 | 0.0265 | No |
| 20 | IL7R | na | 3715 | 0.270 | 0.0308 | No |
| 21 | PDGFRA | na | 4146 | 0.255 | 0.0232 | No |
| 22 | TNFRSF19 | na | 4189 | 0.253 | 0.0268 | No |
| 23 | FLT3 | na | 4865 | 0.230 | 0.0116 | No |
| 24 | CCR10 | na | 4891 | 0.229 | 0.0152 | No |
| 25 | CXCR2 | na | 5077 | 0.223 | 0.0141 | No |
| 26 | IL3 | na | 5142 | 0.221 | 0.0164 | No |
| 27 | CCR6 | na | 5158 | 0.220 | 0.0202 | No |
| 28 | IL5RA | na | 5241 | 0.217 | 0.0219 | No |
| 29 | GH1 | na | 5883 | 0.196 | 0.0071 | No |
| 30 | TNFSF13B | na | 6053 | 0.191 | 0.0058 | No |
| 31 | IL2RB | na | 6899 | 0.165 | -0.0156 | No |
| 32 | GHR | na | 7932 | 0.138 | -0.0429 | No |
| 33 | TPO | na | 7938 | 0.138 | -0.0404 | No |
| 34 | IL20RA | na | 7991 | 0.136 | -0.0393 | No |
| 35 | CCR3 | na | 8240 | 0.131 | -0.0441 | No |
| 36 | IFNL2 | na | 8303 | 0.129 | -0.0434 | No |
| 37 | CCL17 | na | 8406 | 0.127 | -0.0439 | No |
| 38 | PPBP | na | 8715 | 0.120 | -0.0506 | No |
| 39 | IFNE | na | 9115 | 0.111 | -0.0601 | No |
| 40 | TNFSF8 | na | 9137 | 0.110 | -0.0586 | No |
| 41 | IL13 | na | 9160 | 0.110 | -0.0571 | No |
| 42 | ACVR2B | na | 9239 | 0.108 | -0.0573 | No |
| 43 | TNFSF9 | na | 9314 | 0.107 | -0.0574 | No |
| 44 | TGFBR2 | na | 9513 | 0.102 | -0.0612 | No |
| 45 | FLT4 | na | 9545 | 0.102 | -0.0602 | No |
| 46 | FASLG | na | 9725 | 0.098 | -0.0635 | No |
| 47 | TNFRSF13B | na | 9844 | 0.096 | -0.0651 | No |
| 48 | PDGFA | na | 9861 | 0.095 | -0.0638 | No |
| 49 | CCL15 | na | 9912 | 0.094 | -0.0634 | No |
| 50 | BMPR2 | na | 10089 | 0.091 | -0.0668 | No |
| 51 | IL23R | na | 10107 | 0.090 | -0.0656 | No |
| 52 | IL18R1 | na | 10171 | 0.089 | -0.0657 | No |
| 53 | CCR4 | na | 10538 | 0.083 | -0.0748 | No |
| 54 | INHBC | na | 10667 | 0.080 | -0.0770 | No |
| 55 | CCL23 | na | 11184 | 0.072 | -0.0906 | No |
| 56 | TNFSF15 | na | 11484 | 0.067 | -0.0980 | No |
| 57 | IL17RB | na | 11732 | 0.064 | -0.1039 | No |
| 58 | TGFB2 | na | 11803 | 0.063 | -0.1047 | No |
| 59 | ACVRL1 | na | 11851 | 0.062 | -0.1049 | No |
| 60 | GH2 | na | 12078 | 0.059 | -0.1104 | No |
| 61 | CXCR5 | na | 12403 | 0.055 | -0.1187 | No |
| 62 | PF4V1 | na | 12586 | 0.052 | -0.1230 | No |
| 63 | TNFRSF14 | na | 13238 | 0.044 | -0.1411 | No |
| 64 | IL22 | na | 13346 | 0.043 | -0.1433 | No |
| 65 | PLEKHO2 | na | 13361 | 0.043 | -0.1429 | No |
| 66 | CCL11 | na | 13754 | 0.039 | -0.1536 | No |
| 67 | XCL1 | na | 14545 | 0.030 | -0.1759 | No |
| 68 | PDGFRB | na | 14565 | 0.029 | -0.1759 | No |
| 69 | IFNB1 | na | 14716 | 0.028 | -0.1797 | No |
| 70 | IL13RA1 | na | 15119 | 0.023 | -0.1909 | No |
| 71 | CCL4L2 | na | 15123 | 0.023 | -0.1906 | No |
| 72 | IFNA2 | na | 15155 | 0.023 | -0.1910 | No |
| 73 | FLT1 | na | 15419 | 0.020 | -0.1983 | No |
| 74 | TSLP | na | 15472 | 0.020 | -0.1994 | No |
| 75 | IL21R | na | 16118 | 0.013 | -0.2179 | No |
| 76 | IL18 | na | 16210 | 0.013 | -0.2203 | No |
| 77 | CNTFR | na | 16226 | 0.012 | -0.2205 | No |
| 78 | XCR1 | na | 16622 | 0.008 | -0.2318 | No |
| 79 | PRL | na | 16840 | 0.006 | -0.2380 | No |
| 80 | CCL18 | na | 17170 | 0.003 | -0.2474 | No |
| 81 | IL20RB | na | 17221 | 0.002 | -0.2489 | No |
| 82 | EGF | na | 17351 | 0.001 | -0.2526 | No |
| 83 | CD70 | na | 17506 | -0.001 | -0.2570 | No |
| 84 | CCL1 | na | 17639 | -0.002 | -0.2608 | No |
| 85 | IL15RA | na | 18009 | -0.006 | -0.2714 | No |
| 86 | ACVR1B | na | 18273 | -0.009 | -0.2789 | No |
| 87 | CCL26 | na | 18307 | -0.010 | -0.2796 | No |
| 88 | VEGFB | na | 18662 | -0.013 | -0.2897 | No |
| 89 | IL6R | na | 18964 | -0.017 | -0.2981 | No |
| 90 | CXCL5 | na | 19002 | -0.017 | -0.2988 | No |
| 91 | CCL24 | na | 19163 | -0.019 | -0.3031 | No |
| 92 | IL17RA | na | 19239 | -0.020 | -0.3049 | No |
| 93 | CCL16 | na | 19397 | -0.022 | -0.3090 | No |
| 94 | HGF | na | 19424 | -0.022 | -0.3094 | No |
| 95 | EGFR | na | 19596 | -0.024 | -0.3139 | No |
| 96 | IL11RA | na | 19989 | -0.028 | -0.3247 | No |
| 97 | TNFRSF10A | na | 20067 | -0.029 | -0.3264 | No |
| 98 | CCL8 | na | 20722 | -0.037 | -0.3447 | No |
| 99 | CTF1 | na | 20992 | -0.040 | -0.3517 | No |
| 100 | CCL27 | na | 21088 | -0.041 | -0.3537 | No |
| 101 | EDA2R | na | 21219 | -0.043 | -0.3566 | No |
| 102 | EDAR | na | 21285 | -0.043 | -0.3577 | No |
| 103 | CRLF2 | na | 21344 | -0.044 | -0.3585 | No |
| 104 | CXCL14 | na | 21482 | -0.046 | -0.3616 | No |
| 105 | TNFSF10 | na | 21562 | -0.047 | -0.3631 | No |
| 106 | BMPR1A | na | 21644 | -0.047 | -0.3645 | No |
| 107 | CCL19 | na | 21660 | -0.048 | -0.3640 | No |
| 108 | TNFRSF10B | na | 21662 | -0.048 | -0.3632 | No |
| 109 | IL19 | na | 21806 | -0.049 | -0.3664 | No |
| 110 | BMP7 | na | 21836 | -0.050 | -0.3663 | No |
| 111 | TNFSF11 | na | 22090 | -0.053 | -0.3726 | No |
| 112 | IFNAR1 | na | 22120 | -0.053 | -0.3724 | No |
| 113 | TNFRSF1A | na | 22241 | -0.055 | -0.3749 | No |
| 114 | CXCL16 | na | 22313 | -0.056 | -0.3759 | No |
| 115 | ACVR2A | na | 22361 | -0.056 | -0.3762 | No |
| 116 | CCR1 | na | 22388 | -0.057 | -0.3758 | No |
| 117 | CSF3R | na | 22572 | -0.059 | -0.3800 | No |
| 118 | IL12B | na | 22622 | -0.060 | -0.3803 | No |
| 119 | EPO | na | 22797 | -0.062 | -0.3842 | No |
| 120 | CCR8 | na | 22954 | -0.065 | -0.3875 | No |
| 121 | IL5 | na | 23454 | -0.072 | -0.4006 | No |
| 122 | KIT | na | 23499 | -0.073 | -0.4005 | No |
| 123 | LTBR | na | 23537 | -0.073 | -0.4001 | No |
| 124 | CCR5 | na | 23558 | -0.074 | -0.3993 | No |
| 125 | CXCL12 | na | 23758 | -0.077 | -0.4036 | No |
| 126 | FLT3LG | na | 24043 | -0.081 | -0.4103 | No |
| 127 | LTA | na | 24133 | -0.083 | -0.4113 | No |
| 128 | IL2RA | na | 24251 | -0.085 | -0.4131 | No |
| 129 | TNFRSF12A | na | 24430 | -0.088 | -0.4166 | No |
| 130 | IL6ST | na | 24876 | -0.096 | -0.4277 | No |
| 131 | TNFSF14 | na | 24897 | -0.096 | -0.4264 | No |
| 132 | IFNGR1 | na | 24988 | -0.098 | -0.4272 | No |
| 133 | CSF1 | na | 25005 | -0.098 | -0.4258 | No |
| 134 | VEGFA | na | 25195 | -0.102 | -0.4293 | No |
| 135 | TNFRSF1B | na | 25465 | -0.107 | -0.4351 | No |
| 136 | KITLG | na | 25692 | -0.111 | -0.4395 | No |
| 137 | TNFSF13 | na | 25693 | -0.111 | -0.4374 | No |
| 138 | OSM | na | 25761 | -0.112 | -0.4372 | No |
| 139 | IL25 | na | 25879 | -0.114 | -0.4384 | No |
| 140 | IL2RG | na | 26016 | -0.117 | -0.4402 | No |
| 141 | IL1A | na | 26246 | -0.122 | -0.4445 | No |
| 142 | CXCL13 | na | 26404 | -0.125 | -0.4467 | No |
| 143 | CCR2 | na | 26723 | -0.133 | -0.4533 | No |
| 144 | IL12RB2 | na | 26833 | -0.136 | -0.4539 | No |
| 145 | IL12RB1 | na | 27050 | -0.140 | -0.4575 | Yes |
| 146 | CCL14 | na | 27083 | -0.141 | -0.4558 | Yes |
| 147 | TGFBR1 | na | 27137 | -0.142 | -0.4546 | Yes |
| 148 | IFNAR2 | na | 27194 | -0.144 | -0.4535 | Yes |
| 149 | IFNGR2 | na | 27386 | -0.149 | -0.4562 | Yes |
| 150 | CLCF1 | na | 27455 | -0.150 | -0.4553 | Yes |
| 151 | IL10RB | na | 27475 | -0.151 | -0.4530 | Yes |
| 152 | TNFSF12 | na | 27676 | -0.156 | -0.4558 | Yes |
| 153 | TNFRSF17 | na | 27772 | -0.159 | -0.4556 | Yes |
| 154 | CCL13 | na | 27846 | -0.161 | -0.4546 | Yes |
| 155 | TNFRSF10C | na | 27907 | -0.162 | -0.4533 | Yes |
| 156 | IL2 | na | 27917 | -0.162 | -0.4505 | Yes |
| 157 | FAS | na | 27948 | -0.163 | -0.4482 | Yes |
| 158 | LIFR | na | 27949 | -0.163 | -0.4451 | Yes |
| 159 | CXCL11 | na | 27958 | -0.164 | -0.4422 | Yes |
| 160 | CCL3L3 | na | 28062 | -0.166 | -0.4421 | Yes |
| 161 | CCL28 | na | 28142 | -0.169 | -0.4411 | Yes |
| 162 | IL10 | na | 28370 | -0.176 | -0.4444 | Yes |
| 163 | TNFSF4 | na | 28376 | -0.176 | -0.4412 | Yes |
| 164 | CSF1R | na | 28378 | -0.176 | -0.4379 | Yes |
| 165 | IFNA7 | na | 28473 | -0.179 | -0.4372 | Yes |
| 166 | CCL2 | na | 28523 | -0.180 | -0.4352 | Yes |
| 167 | CXCL2 | na | 28621 | -0.183 | -0.4345 | Yes |
| 168 | IL22RA1 | na | 28720 | -0.186 | -0.4338 | Yes |
| 169 | TNFRSF11B | na | 28793 | -0.189 | -0.4323 | Yes |
| 170 | MET | na | 28809 | -0.189 | -0.4292 | Yes |
| 171 | RELT | na | 28836 | -0.190 | -0.4263 | Yes |
| 172 | LEPR | na | 28912 | -0.192 | -0.4248 | Yes |
| 173 | IL20 | na | 29027 | -0.196 | -0.4244 | Yes |
| 174 | TNFRSF4 | na | 29057 | -0.196 | -0.4215 | Yes |
| 175 | CSF3 | na | 29235 | -0.202 | -0.4228 | Yes |
| 176 | CCR9 | na | 29240 | -0.202 | -0.4191 | Yes |
| 177 | AMH | na | 29285 | -0.203 | -0.4165 | Yes |
| 178 | IFNA8 | na | 29291 | -0.203 | -0.4128 | Yes |
| 179 | TNFRSF10D | na | 29448 | -0.208 | -0.4133 | Yes |
| 180 | CXCL6 | na | 29519 | -0.210 | -0.4114 | Yes |
| 181 | ACVR1 | na | 29917 | -0.222 | -0.4187 | Yes |
| 182 | PDGFB | na | 30041 | -0.227 | -0.4179 | Yes |
| 183 | TNFRSF21 | na | 30114 | -0.230 | -0.4156 | Yes |
| 184 | IL9R | na | 30120 | -0.230 | -0.4114 | Yes |
| 185 | TGFB1 | na | 30144 | -0.231 | -0.4077 | Yes |
| 186 | CD40LG | na | 30278 | -0.236 | -0.4070 | Yes |
| 187 | IL1B | na | 30302 | -0.237 | -0.4032 | Yes |
| 188 | IL1RAP | na | 30401 | -0.241 | -0.4015 | Yes |
| 189 | IL23A | na | 30417 | -0.242 | -0.3973 | Yes |
| 190 | IFNLR1 | na | 30449 | -0.243 | -0.3936 | Yes |
| 191 | CCL21 | na | 30451 | -0.243 | -0.3890 | Yes |
| 192 | IL7 | na | 30496 | -0.245 | -0.3856 | Yes |
| 193 | IFNW1 | na | 30630 | -0.250 | -0.3847 | Yes |
| 194 | CCL7 | na | 30636 | -0.250 | -0.3801 | Yes |
| 195 | EPOR | na | 30856 | -0.260 | -0.3815 | Yes |
| 196 | TNFRSF6B | na | 30860 | -0.261 | -0.3766 | Yes |
| 197 | CD27 | na | 30904 | -0.263 | -0.3728 | Yes |
| 198 | CXCR6 | na | 30944 | -0.265 | -0.3689 | Yes |
| 199 | CNTF | na | 30965 | -0.266 | -0.3645 | Yes |
| 200 | INHBE | na | 30990 | -0.268 | -0.3601 | Yes |
| 201 | IL24 | na | 31003 | -0.268 | -0.3553 | Yes |
| 202 | LIF | na | 31331 | -0.285 | -0.3594 | Yes |
| 203 | CXCL8 | na | 31337 | -0.285 | -0.3541 | Yes |
| 204 | IFNL1 | na | 31361 | -0.286 | -0.3493 | Yes |
| 205 | TGFB3 | na | 31437 | -0.291 | -0.3460 | Yes |
| 206 | EDA | na | 31497 | -0.295 | -0.3421 | Yes |
| 207 | GDF5 | na | 31574 | -0.299 | -0.3386 | Yes |
| 208 | INHBB | na | 31728 | -0.308 | -0.3372 | Yes |
| 209 | TNF | na | 31738 | -0.308 | -0.3316 | Yes |
| 210 | CXCR3 | na | 31747 | -0.309 | -0.3259 | Yes |
| 211 | TNFRSF9 | na | 31818 | -0.313 | -0.3220 | Yes |
| 212 | IFNA10 | na | 31873 | -0.316 | -0.3176 | Yes |
| 213 | CCL22 | na | 31926 | -0.319 | -0.3130 | Yes |
| 214 | CSF2RB | na | 31957 | -0.321 | -0.3078 | Yes |
| 215 | IL6 | na | 32098 | -0.328 | -0.3056 | Yes |
| 216 | KDR | na | 32219 | -0.336 | -0.3027 | Yes |
| 217 | CCL5 | na | 32237 | -0.337 | -0.2968 | Yes |
| 218 | CCL3 | na | 32264 | -0.338 | -0.2911 | Yes |
| 219 | INHBA | na | 32346 | -0.344 | -0.2869 | Yes |
| 220 | CCL20 | na | 32374 | -0.346 | -0.2811 | Yes |
| 221 | CSF2RA | na | 32416 | -0.349 | -0.2757 | Yes |
| 222 | CD40 | na | 32420 | -0.349 | -0.2691 | Yes |
| 223 | TNFSF18 | na | 32527 | -0.356 | -0.2654 | Yes |
| 224 | PRLR | na | 32623 | -0.362 | -0.2613 | Yes |
| 225 | IL11 | na | 32806 | -0.377 | -0.2594 | Yes |
| 226 | CSF2 | na | 32846 | -0.380 | -0.2533 | Yes |
| 227 | IL4 | na | 32876 | -0.383 | -0.2469 | Yes |
| 228 | TNFRSF8 | na | 32900 | -0.385 | -0.2402 | Yes |
| 229 | IL15 | na | 32921 | -0.387 | -0.2334 | Yes |
| 230 | IL1R1 | na | 32935 | -0.388 | -0.2264 | Yes |
| 231 | IFNG | na | 32943 | -0.389 | -0.2192 | Yes |
| 232 | IL18RAP | na | 33137 | -0.407 | -0.2171 | Yes |
| 233 | TNFRSF11A | na | 33216 | -0.415 | -0.2115 | Yes |
| 234 | CX3CR1 | na | 33225 | -0.415 | -0.2038 | Yes |
| 235 | BMP2 | na | 33393 | -0.437 | -0.2003 | Yes |
| 236 | CXCL3 | na | 33497 | -0.449 | -0.1948 | Yes |
| 237 | CXCR4 | na | 33720 | -0.481 | -0.1921 | Yes |
| 238 | TNFRSF18 | na | 33743 | -0.485 | -0.1835 | Yes |
| 239 | CCR7 | na | 33758 | -0.486 | -0.1746 | Yes |
| 240 | MPL | na | 33774 | -0.489 | -0.1658 | Yes |
| 241 | IL17A | na | 33821 | -0.499 | -0.1576 | Yes |
| 242 | CX3CL1 | na | 33846 | -0.503 | -0.1487 | Yes |
| 243 | IFNK | na | 33874 | -0.507 | -0.1399 | Yes |
| 244 | IFNA21 | na | 33939 | -0.521 | -0.1318 | Yes |
| 245 | IL1R2 | na | 34002 | -0.532 | -0.1235 | Yes |
| 246 | CCL25 | na | 34109 | -0.559 | -0.1159 | Yes |
| 247 | IL22RA2 | na | 34217 | -0.586 | -0.1079 | Yes |
| 248 | VEGFC | na | 34267 | -0.599 | -0.0979 | Yes |
| 249 | PDGFC | na | 34382 | -0.645 | -0.0889 | Yes |
| 250 | CXCR1 | na | 34414 | -0.656 | -0.0774 | Yes |
| 251 | LTB | na | 34462 | -0.678 | -0.0658 | Yes |
| 252 | IL10RA | na | 34468 | -0.683 | -0.0530 | Yes |
| 253 | CXCL1 | na | 34497 | -0.699 | -0.0405 | Yes |
| 254 | OSMR | na | 34510 | -0.705 | -0.0275 | Yes |
| 255 | CXCL9 | na | 34585 | -0.776 | -0.0149 | Yes |
| 256 | CXCL10 | na | 34695 | -0.997 | 0.0010 | Yes |
Table: GSEA details [plain text format]

  

Fig 2: KEGG\_CYTOKINE\_CYTOKINE\_RECEPTOR\_INTERACTION      
 Blue-Pink O' Gram in the Space of the Analyzed GeneSet

  

Fig 3: KEGG\_CYTOKINE\_CYTOKINE\_RECEPTOR\_INTERACTION: Random ES distribution      
 Gene set null distribution of ES for **KEGG\_CYTOKINE\_CYTOKINE\_RECEPTOR\_INTERACTION**
